# Multi-faceted intervention to improve management of antibiotics for children presenting to primary care with acute cough and respiratory tract infection (CHICO): efficient cluster randomised controlled trial

**DOI:** 10.1136/bmj-2022-072488

**Published:** 2023-04-26

**Authors:** Peter S Blair, Grace Young, Clare Clement, P Dixon, Penny Seume, Jenny Ingram, Jodi Taylor, Christie Cabral, Patricia J Lucas, Elizabeth Beech, Jeremy Horwood, Martin Gulliford, Nick A Francis, Sam Creavin, Janet A Lane, Scott Bevan, Alastair D Hay

**Affiliations:** 1 Centre for Academic Child Health, Bristol Medical School, University of Bristol, Bristol, UK; 2 Bristol Trials Centre, Bristol Medical School, University of Bristol, Bristol, UK; 3 Nuffield Department of Primary Care Health Sciences, University of Oxford, Oxford, UK; 4 Centre for Academic Primary Care, Bristol Medical School, University of Bristol, Bristol, UK; 5 School for Policy Studies, University of Bristol, Bristol, UK; 6 NHS England and NHS Improvement South West, Bath, UK; 7 King’s College London, School of Population and Life Course Sciences London, UK^8^Primary Care Research Centre, School of Primary Care Population Sciences and Medical Education, University of Southampton, Southampton, UK

## Abstract

**Objective:**

To assess whether an easy-to-use multifaceted intervention for children presenting to primary care with respiratory tract infections would reduce antibiotic dispensing, without increasing hospital admissions for respiratory tract infection.

**Design:**

Two arm randomised controlled trial clustered by general practice, using routine outcome data, with qualitative and economic evaluations.

**Setting:**

English primary care practices using the EMIS electronic medical record system.

**Participants:**

Children aged 0-9 years presenting with respiratory tract infection at 294 general practices, before and during the covid-19 pandemic.

**Intervention:**

Elicitation of parental concerns during consultation; a clinician focused prognostic algorithm to identify children at very low, normal, or elevated 30 day risk of hospital admission accompanied by antibiotic prescribing guidance; and a leaflet for carers including safety netting advice.

**Main outcome measures:**

Rate of dispensed amoxicillin and macrolide antibiotics (superiority comparison) and hospital admissions for respiratory tract infection (non-inferiority comparison) for children aged 0-9 years over 12 months (same age practice list size as denominator).

**Results:**

Of 310 practices needed, 294 (95%) were randomised (144 intervention and 150 controls) representing 5% of all registered 0-9 year olds in England. Of these, 12 (4%) subsequently withdrew (six owing to the pandemic). Median intervention use per practice was 70 (by a median of 9 clinicians). No evidence was found that antibiotic dispensing differed between intervention practices (155 (95% confidence interval 138 to 174) items/year/1000 children) and control practices (157 (140 to 176) items/year/1000 children) (rate ratio 1.011, 95% confidence interval 0.992 to 1.029; P=0.25). Pre-specified subgroup analyses suggested reduced dispensing in intervention practices with fewer prescribing nurses, in single site (compared with multisite) practices, and in practices located in areas of lower socioeconomic deprivation, which may warrant future investigation. Pre-specified sensitivity analysis suggested reduced dispensing among older children in the intervention arm (P=0.03). A post hoc sensitivity analysis suggested less dispensing in intervention practices before the pandemic (rate ratio 0.967, 0.946 to 0.989; P=0.003). The rate of hospital admission for respiratory tract infections in the intervention practices (13 (95% confidence interval 10 to 18) admissions/1000 children) was non-inferior compared with control practices (15 (12 to 20) admissions/1000 children) (rate ratio 0.952, 0.905 to 1.003).

**Conclusions:**

This multifaceted antibiotic stewardship intervention for children with respiratory tract infections did not reduce overall antibiotic dispensing or increase respiratory tract infection related hospital admissions. Evidence suggested that in some subgroups and situations (for example, under non-pandemic conditions) the intervention slightly reduced prescribing rates but not in a clinically relevant way.

**Trial registration:**

ISRCTN11405239ISRCTN registry ISRCTN11405239

## Introduction

Unnecessary use of antibiotics is associated with the needless development and proliferation of antimicrobial resistance.^1^ ^2^ Around 80% of antibiotics prescribed for human consumption are prescribed in primary care,^3^ and around 50% of antibiotic prescribing in this setting has been estimated to be unnecessary.^4^ Children with respiratory tract infections are the single most frequent patient group to make use of primary care and receive antibiotics,^5^ with clinical uncertainty about prognosis leading to defensive antibiotic use.^6^ Although prescribing for uncomplicated respiratory tract infection has declined in England over the past decade, more than a third of children were still given antibiotics for these infections.^7^

Previous qualitative research has also shown that clinicians prescribe antibiotics “just in case” to mitigate perceived risk of future hospital admission and complications,^8^ ^9^ that parents want better information on the signs and symptoms of serious respiratory tract infections and when to consult,^10^ and that parents also want more useful advice on home management of symptoms.^6^ We hypothesised that improved identification of children at very low risk of future hospital admission might help to reduce clinical uncertainty and that, combined with improved parent communications, this might reduce antibiotic use.

In an attempt to reduce “just in case” prescribing, we previously developed and internally validated the STARWAVe prognostic algorithm, which uses seven clinical characteristics to stratify children’s risk of hospital admission in the following 30 days into “very low,” “normal,” and “high” risk (bootstrapped area under the receiver operating characteristic curve 0.81). Children in the very low risk stratum have a 0.3% (95% confidence interval 0.2% to 0.4%) post-test probability of needing hospital treatment in the following 30 days and thus are unlikely to need antibiotics.^11^ As no datasets known to contain STARWAVe variables were available for external validation, we considered that the next step was to investigate clinical effectiveness. The algorithm was combined with tools to improve patient-doctor communication and parent information,^12^ following the Medical Research Council’s framework guidelines for development of complex interventions.^13^ We then conducted an individual patient randomised feasibility trial of the resulting intervention in a similar practice population, concluding that a future trial should reduce the time needed in the consultation to recruit patients and differential recruitment of less unwell patients between study arms.^14^ The trial we now report overcomes these problems by randomising general practice clusters (thereby avoiding individual patient recruitment) and using routinely collected aggregate outcome data (thereby avoiding the need for parental consent, as well as being more resource efficient).^15^ The intervention was also embedded within the general practice electronic medical record system. The aim of this study was to assess whether a refined, multifaceted intervention could reduce antibiotic dispensing among children aged 0-9 years presenting with cough and respiratory tract infection, without increasing respiratory tract infection related hospital admissions.

## Methods

### Study design

The CHICO (CHIldren’s COugh) randomised controlled trial was an efficient, pragmatic, open label, two arm (intervention versus usual care) trial of children aged 0-9, clustered by primary care practices in England. The trial included an embedded qualitative study that will be reported in detail elsewhere and a health economic analysis. The CHICO trial randomised practices between October 2018 and October 2020, with a trial data collection period from November 2018 to September 2021, thus including the covid-19 pandemic that began in March 2020 and was still ongoing in September 2021. We recruited practices by using clinical commissioning groups (CCGs; renamed integrated care boards from 1 July 2022) and the National Institute for Health and Care Research’s Clinical Research Network. We did an internal pilot in three CCGs initially to identify any problems with the intervention. This lasted three months and included a further four CCGs to help to establish best practice for recruiting and communicating with practices before widening to the remaining CCGs. The trial protocol has been published.^16^

### Recruitment

Recruitment comprised two elements. Firstly, as CCGs routinely collected the co-primary outcome of hospital admission for respiratory tract infections, we endeavoured to recruit CCGs to the study. We targeted CCG recruitment at those with 15 or more practices using the EMIS patient record system (used in 56% of English practices).^17^ Secondly, practices were recruited from within participating CCGs by enlisting the help of the 15 clinical research networks across England. We excluded practices if they were not using the EMIS system, were participating in any potentially confounding concurrent intervention studies (for example, antimicrobial stewardship), or were merging, or planning to merge, with another practice. Once a practice had agreed to take part in the trial and completed their baseline questionnaire, it was randomised. The trial manager used the Bristol Randomised Trials Collaboration clinical trials unit randomisation system to randomise practices, stratified by CCG, using a one to one allocation, to either the intervention or control (usual care) arm, and using minimisation to balance list size and past dispensing rate, in 0-9 year olds, in the 12 months before randomisation. Practices used the intervention over a 12 month period from randomisation. The clinicians from practices randomised to the control arm were asked to treat children presenting with cough or respiratory tract infection as they usually would. A practice champion was appointed at each intervention practice to distribute training materials within the practice, coordinate training of prescribing staff, encourage all clinicians to use the intervention appropriately, and report from the EMIS system how many times the intervention was used.

### The intervention

The intervention consisted of eliciting explicit concerns from carers during consultation, a clinician focused algorithm to predict risk (low, normal, or elevated) of hospital admission within 30 days for children consulting with a respiratory tract infection along with antibiotic prescribing guidance, and a carer focused personalised printout recording decisions made at the consultation, covering common concerns and providing safety netting information, which was based on a leaflet co-designed with parents.^18^ We sent intervention practices instructions including screen shots on how to install the intervention’s algorithm on the EMIS system. The algorithm included seven predictors, two of which (age of patient and history of asthma) were already available for automatic entry; the other five predictors (short illness duration, temperature, intercostal or subcostal recession on examination, wheeze, and moderate or severe vomiting) were entered during consultation. Table 1 provides the resulting comment associated with each score (each predictor scores one point). The training package for clinicians emphasised that the primary purpose of the algorithm was to identify the large proportion of children (69%) who have a very low risk of hospital admission and so could be safely managed at home. When a child in the eligible age range attended, the healthcare professional received a “soft” (that is, a reminder) screen alert (Quality and Outcomes Framework prompt style) asking if the child was presenting with a respiratory tract infection. The alert gave the option of opening the CHICO intervention template; alternatively, entry of specific respiratory tract infection codes triggered the launch of the template during the consultation. Each use of the template was automatically recorded using an EMIS code. Given the nature of the trial intervention, practices were not blinded to their allocation. The trial manager, administrative staff, and statistician were the only members of the trial management group who were unblinded.

**Table 1:** Clinician advice associated with the algorithm output*

CHICO result	Pop-up text
Low risk group	Very reassuring CHICO score: 0 or 1 CHICO predictors: >99.6% of children will recover from this illness with home care. Consider a no or delayed antibiotic prescribing strategy. CHICO leaflet and letter covers common concerns and safety netting advice
Average risk group	Reassuring CHICO score: 2 or 3 CHICO predictors: >98% of children will recover from this illness with home care. Consider no or delayed antibiotic prescribing strategy. CHICO leaflet and letter covers common concerns and safety netting advice
Elevated risk group	Safety netting needed: ≥4 CHICO predictors: This is more than average, but >87% of children will still recover from this illness with home care. Highlight safety netting advice in CHICO leaflet

* As presented in Seume et al, 2021.^15^

### Outcomes

The co-primary outcomes for children aged 0-9 years over a 12 month period were the rate of dispensed amoxicillin and macrolide items prescribed, for all indications (superiority comparison) collected routinely by NHSBSA ePACT2 (Electronic Prescribing Analysis and Cost) system,^19^ and the rate of hospital admission for respiratory tract infection (non-inferiority comparison) routinely collected by CCGs. As no children were directly consented/recruited into the trial, we used proxy measures, for which the denominator was all 0-9 year old children registered at each practice. We collected baseline data on the characteristics of the practice and follow-up data after 12 months. Table 2 lists primary and secondary outcomes (including subgroup analyses).

**Table 2:** Primary and secondary outcomes*

Outcome No	Description
**Primary outcomes**	
P1	Rate of amoxicillin and macrolide items dispensed, calculated using number of items dispensed to 0-9 year olds and number of children aged 0-9 registered at each practice over 12 month period (testing for superiority). Number of items dispensed, for all indications, was used as proxy measure owing to limitations of routine data
P2	Rate of hospital admissions for RTI, calculated using number of admissions for RTI among children aged 0-9 years and number of children aged 0-9 registered at each practice over 12 month period (testing for non-inferiority)
**Secondary outcomes**	
S1	Emergency department attendance rates for RTI, calculated using number of attendances for RTI among children aged 0-9 years and number of children aged 0-9 registered at each practice over 12 month period
S2	Exploration into usage of intervention, in terms of both usage over 12 month period and seasonality, and effects it has on primary outcome P1
S3	Between arm comparison of mean NHS costs in cost-consequence analysis (health economics)
S4	Acceptability of intervention and variation in use determined by qualitative interviews with clinicians
S5	Comparison of primary outcome P1 stratified by categorisation of proportion of locums (median) used over 12 months practice is in study
S6	Comparison of primary outcome P1 stratified by categorisation of proportion of nurses (median) used over 12 months practice is in study. This was originally planned as dichotomisation of practices into those with GP prescribers only and those with nurse or other prescribers as well. However, given that most practices had nurse prescribers, it seemed sensible to look at nurses as proportion of staff
S7	Comparison of primary outcome P1 stratified by categorisation of practice dispensing rates taken from 12 months before data collection from each practice
S8	Comparison of primary outcome P1 dichotomised by practices with one site only and those with multiple sites
S9	Comparison of primary outcome P1 dichotomised by practices with follow-up periods before covid-19 pandemic and those with follow-up months on or after March 2020
S10	Comparison of primary outcome P1 dichotomised by practices with high level of deprivation versus those with low level of deprivation

GP=general practitioner; RTI=respiratory tract infection.

* As presented in Seume et al, 2021.^15^

### Serious adverse events

The trial was considered low risk, so serious adverse events were reported only if fatal or serious and potentially related to trial participation. As one of the outcomes for the trial was hospital admission, we expected that some participants would be admitted to hospital (owing to a deterioration of their underlying illness, for example) so this was not subject to expedited reporting.

### Sample size

The trial consisted of two co-primary outcomes, so we used a two sided α of 0.025 to take account of this. Both sample size calculations used a comparison of two proportions and assumed 90% power, an intra-cluster correlation coefficient of 0.03, a coefficient of variation of 0.65, and an estimated 750 children (registered 0-9 year olds) per practice. We estimated that 33 items would be dispensed and one hospital admission would occur per 100 children. To detect a 10% reduction, to 29 items, in dispensing data (superiority) and no more than an absolute 1% increase, to two, in hospital admissions (non-inferiority), we needed 155 practices per arm. We could conclude non-inferiority if the rate ratio, comparing the intervention arm with the control, had an upper confidence interval ≤1.01.

### Analytical methods

A full CHICO statistical, health economic, and qualitative analysis plan was developed and agreed by an independent Trial Steering Committee and Data Monitoring Committee.^20^ All primary and secondary analyses were conducted on an intention-to-treat basis. General practices were the unit of randomisation/analysis in this cluster randomised trial, with the randomisation stratified by CCGs. Any variation between CCGs was accommodated by using CCG level random effects—that is, a two level model. We used mixed effects Poisson regression models to derive the rate ratio for both co-primary outcomes, including the practice list size (of 0-9 year old children) as the exposure/time variable, the baseline rate as a covariate, and random effects at the CCG level. We used the dispensing record of the practices in the 12 months before randomisation as a minimisation variable, thus balancing the dispensing records at baseline. We adjusted for this, in the primary analysis of dispensing rates, to resolve any residual difference. Although rates are described throughout, these are a calculation of dispensed items divided by list size of 0-9 year old children. Therefore, to aid interpretation in the text, rates have been converted to number per year per 1000 children. We analysed the hospital admission outcome in a similar way to the dispensing outcome but tested for non-inferiority, whereby a difference of no more than 1% higher in the intervention arm indicated non-inferiority, with emphasis placed on the confidence interval. We adjusted for baseline hospital admission rates in this analysis. We compared baseline characteristics descriptively, between the arms, and planned an adjusted sensitivity analysis if these differed by more than 10% (categorical variables), half a standard deviation, or half an interquartile range (continuous variables). We calculated the index of multiple deprivation by using the postcode of the practice in which the practice champion was based, using online routine data to group the practices into tenths of deprivation; data were available from 2019.^21^ Other sensitivity analyses have been described fully in the supplementary material. Subgroup analyses were pre-specified and investigated the treatment effect within subgroups of practices (dichotomised). To determine whether the treatment effect differed across the levels of the subgroup (for example, proportion of locums), we used likelihood ratio tests to compare models with and without the interaction term included, treating the subgroup of interest as a continuous variable wherever possible. We note that these subgroup analyses do not have sufficient power and should be used as guides for future research rather than confirmatory evidence. We used Stata 17.0 for all analyses and described the results in terms of strength of evidence rather than significance. We compared mean differences in NHS costs between arms by using two way mixed effect linear regression.

### Patient and public involvement

The CHICO intervention was developed in collaboration with both a Parent Advisory Group and a Clinician and Practitioner Advisory Group, which we met several times during the final year of the CHICO feasibility trial. The Parent Advisory Group thought that conducting a national study and using a whole practice intervention were important and that using a prediction tool on the computer during consultation would provide reassurance. They strongly endorsed and encouraged us to use the parent leaflet. Patient and public involvement was maintained throughout the main trial through a group of three parents, two of whom (in rotation) attended and contributed to all the Trial Steering Committee meetings, including the final results reveal meeting. As the intervention focus was on general practices and not on recruiting individual patients, we made extensive contact with the Clinician and Practitioner Advisory Group during the trial period. We sought advice on the use of alert style and trigger codes in a consultation, the format of the template, the personalised letter, and questionnaires used to collect the data. We also ran some “think aloud” sessions with several general practitioners to see the intervention in action in EMIS and gather their thoughts about any problems or changes needed. The findings were disseminated to the patient and public involvement group and Trial Steering Committee members for comment and presented at primary care conferences, and a summary will be fed back to practices as the data are published.

## Results

### Recruitment and ascertainment

**Fig 1:**
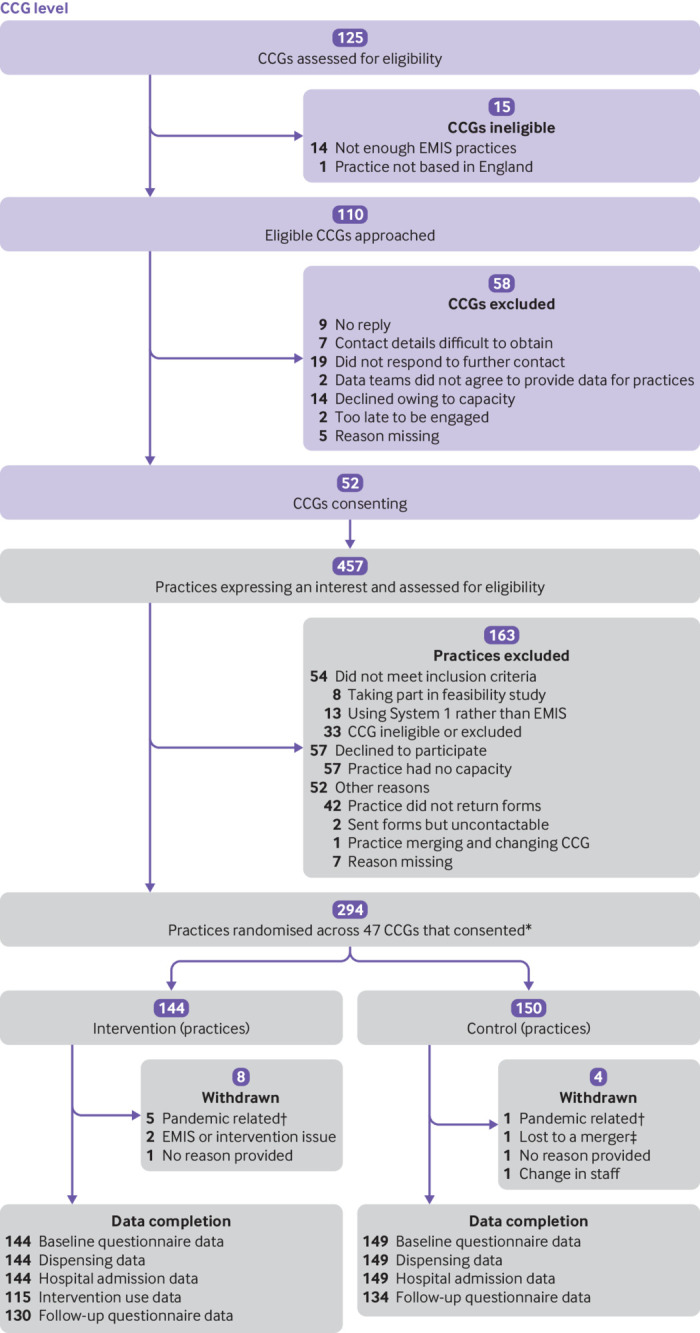
CHICO trial Consolidated Standards of Reporting Trials flowchart. CCG=clinical commissioning group; EMIS=electronic health record system. *One practice randomised was made up of two practices that work closely and were due to merge. †Reasons include lack of staff, lack of resources, and lack of consultations for children with cough. ‡Two practices in control arm merged, so outcome data were compiled into one practice

Of approximately 200 CCGs in England in October 2018, 110 were assessed as being eligible (≥15 practices using EMIS); 52 consented to take part, and 47 provided at least one practice (fig 1). Recruitment took 24 rather than 12 months, continuing to October 2020 (owing to slow response of some CCGs and effects of the covid-19 pandemic). Of the 310 practices needed, 294 (95%) were recruited (144 intervention and 150 controls), representing 336 496 registered 0-9 year-olds (5% of all 0-9 year old children in England). Of the 294 practices, 12 (4%) subsequently withdrew (six owing to the pandemic). Four serious adverse events (three intervention; one control) were reported, none of them related to the intervention.

### Baseline characteristics

The two randomised arms were well balanced with respect to baseline characteristics (table 3) and did not meet pre-specified criteria for sensitivity analysis. Of the practices randomised, 62% had a high list size (of 0-9 year old children) relative to other practices in their CCG, suggesting that smaller practices were less likely to be recruited. Similarly, randomised practices had lower baseline dispensing rates and were located in areas of higher socioeconomic deprivation.

**Table 3:** Practice level baseline characteristics, by arm. Values are numbers (percentages) unless stated otherwise

Characteristic	Intervention			Control	
	No*	Value		No*	Value
**Randomisation variables** **†**					
High list size (0-9 year olds), relative to others in their CCG	144	92 (64)		150	91 (61)
High dispensing rate, relative to others in their CCG	144	60 (42)		150	66 (44)
**Baseline practice level** **‡**					
Median (IQR) list size§: all ages 0-9	144	975 (701-1422)		150	997 (645-1375)
Median (IQR) list size§: age 0-4 epoch	144	480 (339-682)		150	474 (308-62)
Median (IQR) list size§: age 5-9 epoch	144	485 (349-780)		150	515 (334-723)
Median (IQR) dispensing rate, per 100 list size (0-9 year olds)	144	19.0 (14.3-24.9)		150	17.7 (14.3-23.9)
CHICO leaflet in use at practice	142	12 (8)		142	10 (7)
Median (IQR) distance to nearest children’s emergency department, miles	142	4.7 (2.3-9.0)		145	4.0 (2.2-11.5)
IMD fifth based on practice postcode:					
1 (most deprived)	144	37 (26)		150	40 (27)
2		29 (20)			31 (21)
3		31 (22)			29 (19)
4		28 (19)			26 (17)
5 (least deprived)		19 (13)			24 (16)
Median (IQR) income deprivation affecting children index score	144	0.15 (0.08-0.25)		150	0.15 (0.08-0.26)
**Practice staff**					
Median (IQR) No of general practitioners	143	6.0 (4.0-10.0)		147	6.0 (4.0-9.0)
Median (IQR) No of salaried nurses	114	2.0 (1.0-4.0)		124	2.0 (1.0-4.0)
Median (IQR) No of sessional nurses	68	0.0 (0.0-1.0)		65	0.0 (0.0-0.0)
Median (IQR) No of pharmacist prescribers	89	1.0 (0.0-1.0)		101	1.0 (0.0-1.0)
Median (IQR) No of locums	102	4.0 (1.0-6.0)		101	3.0 (2.0-6.0)
**Patient management**					
Patient management (not mutually exclusive):					
No triage	141	35 (25)		145	46 (32)
Nurse face to face	141	17 (12)		145	20 (14)
GP face to face	141	47 (33)		145	47 (32)
Receptionist telephone triage	141	44 (31)		145	48 (33)
Nurse telephone triage	141	17 (12)		145	26 (18)
GP telephone triage	141	74 (52)		145	81 (56)
Other¶	141	16 (11)		145	20 (14)
Proportion of RTIs consulted over phone:					
None	142	22 (15)		146	26 (18)
Very few		35 (25)			34 (23)
Some cases		39 (27)			40 (27)
Most cases		32 (23)			29 (20)
Always		14 (10)			17 (12)
Of these, how many are completely dealt with on phone?					
None	138	25 (18)		140	33 (24)
Very few		55 (40)			46 (33)
Some cases		44 (32)			55 (39)
Most cases		13 (9)			5 (4)
Always		1 (1)			1 (1)

CCG=clinical commissioning group; GP=general practitioner; IMD= index of multiple deprivation; IQR=interquartile range; RTI=respiratory tract infection.

* Number of practices with data (denominator).

† When CCGs accepted participation in trial, previous 12 months of list sizes and dispensing figures (in 0-9 year olds) was used to split practices into high and low categories, relative to other practices within their CCG. This information was used in randomisation minimisation process.

‡ Collected from baseline practice questionnaires unless stated otherwise.

§ Collected from NHS digital data.

¶ Includes online (askmyGP), emergency care practitioner, and pharmacist.

### Intervention usage

Across the 121 (84%) intervention practices that provided at least one month of intervention usage data, a total of 11 944 intervention uses were started and recorded (practice median 70 (interquartile range 9-142)). Twenty (17%) practices recorded zero usage over the 12 month period, of which 13 provided data wholly within the covid-19 pandemic period. The median number of clinical staff at intervention practices was 13, and the median number of intervention users per practice was 9 (3-16). Of staff whose job title was captured (n=1339), 74% (n=994) were general practitioners, 14% (187) were nurses, 6% (77) were office staff, 3% (40) were clinicians, 3% (34) were locum general practitioners, and 1% (7) were pharmacists. The baseline and follow-up data collection periods spanned October 2017 to September 2021 and thus included the covid-19 pandemic that began in the spring of 2020. Both use of the intervention and antibiotic dispensing data followed the expected seasonal winter peak until the pandemic, during which the intervention usage dramatically fell (table 4) and seasonal patterns disappeared.

**Table 4:** Median number of times intervention was used, per month, over 12 months of follow-up

Practice	Month of follow-up											
	1	2	3	4	5	6	7	8	9	10	11	12
All intervention practices (n=115)	4	6	4	5	5	2	2	2	0	1	1	0
Pre-covid: completed before March 2020 (n=29)	22	18	11	7	8	4	6	6	7	11	10	7
Partial covid: ≥1 month from March 2020 (n=57)	4	9	7	9	10	6	5	2	0	0	0	0
All covid: randomised on/after March 2020 (n=29)	0	0	0	0	0	0	0	0	0	0	0	0

### Comparison of dispensing rates (co-primary outcome)

The main intention-to-treat analysis (table 5) showed no evidence that the antibiotic dispensing rate in the intervention practices (0.155, 95% confidence interval 0.138 to 0.174) differed from that of the controls (0.157, 0.140 to 0.176), with a rate ratio of 1.011 (95% confidence interval 0.992 to 1.029; P=0.25). On average, this translates into 155 versus 157 items dispensed a year per 1000 children. A rate ratio above 1 signifies that the rate in the intervention practices was higher than in the controls, which may seem to contradict the rates quoted above. Adjustment for baseline dispensing rates caused this change and was influential throughout all analyses.

**Table 5:** Primary and sensitivity analyses for CHICO trial dispensing outcome

Analysis	No (I:C)	Rate* (95% CI)		Adjusted rate ratio (95% CI)†	P value†
		Intervention (I)	Control (C)		
Primary analysis	144:149	0.155 (0.138 to 0.174)	0.157 (0.140 to 0.176)	1.011 (0.992 to 1.029)	0.25
Pre-specified sensitivity analyses:					
Per protocol: excluding non-compliers in intervention arm‡	78:149	0.162 (0.143 to 0.184)	0.161 (0.142 to 0.183)	1.052 (1.029 to 1.076)	<0.001
0-4 year olds only	144:149	0.225 (0.200 to 0.253)	0.225 (0.200 to 0.253)	1.037 (1.014 to 1.060)	0.001
5-9 year olds only	144:149	0.093 (0.083 to 0.103)	0.096 (0.087 to 0.107)	0.965 (0.935 to 0.997)	0.03
Worst case scenario: including “age unknown” as aged 0-9	144:149	0.183 (0.162 to 0.206)	0.184 (0.163 to 0.208)	1.002 (0.986 to 1.018)	0.84
Excluding internal pilot practices (n=48)	117:128	0.150 (0.133 to 0.169)	0.153 (0.136 to 0.183)	1.026 (1.006 to 1.048)	0.012
Adjusting for No of months affected by covid-19 (0-12)§	144:149	0.155 (0.138 to 0.174)	0.157 (0.140 to 0.176)	1.002 (0.984 to 1.021)	0.79
Post hoc sensitivity analyses:					
Complier average causal effect analysis¶	144:145			1.023 (0.897 to 1.166)	0.74
Excluding individual practice months of data from March 2020**	105:121	0.231 (0.208 to 0.257)	0.249 (0.224 to 0.277)	0.967 (0.946 to 0.989)	0.003
Amoxicillin items only (macrolides excluded)	144:149	0.120 (0.107 to 0.136)	0.124 (0.110 to 0.139)	0.994 (0.974 to 1.014)	0.56
Adjusting for random effects at PCN level	144:149	0.150 (0.141 to 0.161)	0.151 (0.141 to 0.162)	0.942 (0.916 to 0.969)	<0.001
Adjusting for delayed start (number of months)††	144:149	0.155 (0.138 to 0.174)	0.157 (0.140 to 0.176)	1.043 (1.023 to 1.063)	<0.001

CCG=clinical commissioning group; CI=confidence interval; PCN=primary care network.

* Rates taken from random effects Poisson regression, incorporating CCG as random effect.

† Random effects Poisson regression, adjusting for baseline dispensing rate and incorporating CCG as random effect.

‡ Excluding practices in intervention arm that did not comply with intervention ([number of uses/list size of children]<0.05) or for which compliance was unknown; excludes practice that merged with another practice that had taken part in control arm.

§ Including numerical variable (0-12) to indicate how many months were affected by covid-19.

¶ Adjusting for baseline rate and number of months affected by covid-19.

** Excluding follow-up months affected by covid-19.

†† Some practices asked to delay their start date and covariate was included to indicate number of months they delayed.

### Comparison of dispensing rates in pre-specified sensitivity analyses

We did five pre-specified sensitivity analyses (table 5) for the dispensing rate primary outcome. The per protocol analysis produced strong evidence of increased dispensing in the intervention arm (162 *v* 161 items a year per 1000 children), although many non-compliant practices joined in the second half of the study, when covid-19 had lowered all dispensing rates leading to a surplus of “low dispensing” practices in the control arm. When we analysed 5-9 year olds only, the dispensing rate was lower in the intervention arm. Conversely, in 0-4 year olds, the dispensing rate was higher in the intervention arm. Other pre-specified sensitivity analyses did not materially change the treatment effect for dispensing rates, including adjusting for the number of months affected by covid-19, assuming “age unknown” was children aged 0-9 years, and excluding the internal pilot practices (n=48).

### Comparison of dispensing rates in post hoc sensitivity analyses

**Fig 2:**
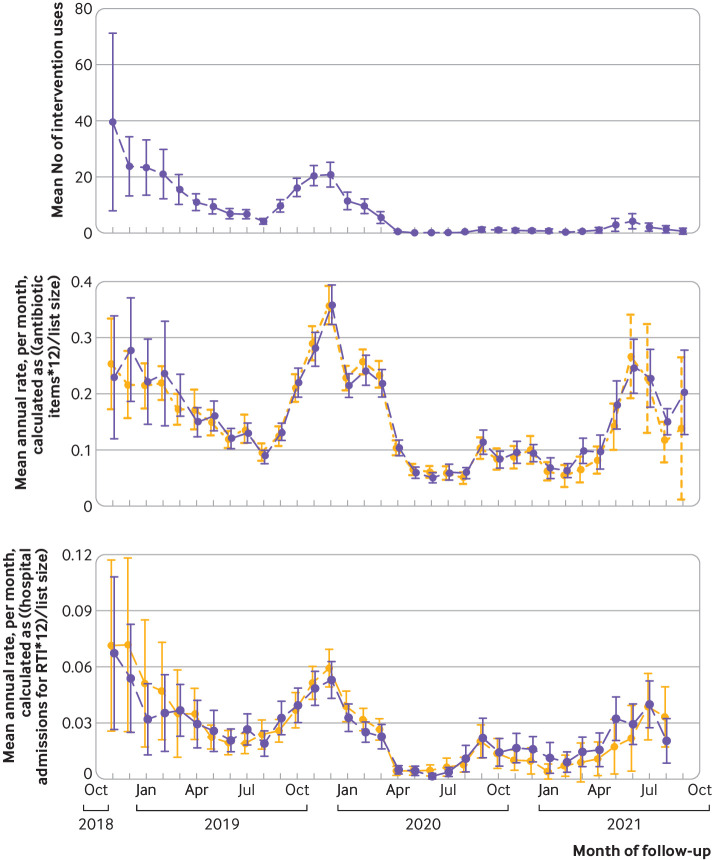
Mean and 95% confidence interval of intervention usage (top), dispensing rates (middle), and hospital admission rates (bottom), over course of CHICO trial follow-up period for intervention and control arms. RTI=respiratory tract infection

We did five post hoc sensitivity analyses (table 5) that were added, largely because of the pandemic. Across all practices the pre-pandemic dispensing rate in the 12 month period before 1 March 2020 was 205 (196 to 215) items per 1000 children, with the usual seasonal patterns (fig 2). In the subsequent 12 months, the dispensing rate halved to 98 (92 to 105) items per 1000 children. We did a complier average causal effect analysis in an attempt to overcome the bias in the per protocol analysis; this produced similar results to the primary outcome. A post hoc sensitivity analysis that excluded any months after March 2020 resulted in a reduced dispensing rate in the intervention arm (table 5). Including the primary care network as the random effects variable, rather than CCG, also led to results that were in favour of the intervention. A delayed start occurred for four practices in the control arm and 36 practices in the intervention arm. Incorporating the number of months delayed as a covariate led to a treatment effect in favour of the control arm. A focus on amoxicillin items only (excluding macrolides) did not materially change dispensing rates.

### Comparison of dispensing rates in subgroup analyses

Some pre-defined subgroup analyses did interact with the treatment effect, with evidence of increased dispensing rates in the intervention arm among practices located in areas with a higher level of deprivation (P=0.004), practices with more than one site (P<0.001), and practices with a higher proportion of prescribing nursing staff (P<0.001) (table 6; supplementary figure A). We found some evidence to suggest that practices with more locums had a treatment effect in favour of the intervention (P=0.005), but, as with many of the other subgroups, the magnitude of effect is difficult to measure in analyses that are underpowered. We found no evidence of an interaction when looking at previous dispensing habits or separating practices into those affected or not affected by covid-19. However, given that some practices may have been affected for one month of follow-up and some for 12 months, this did not successfully capture the effect; thus, we included more post hoc sensitivity analyses, as described in the supplementary material.

**Table 6:** Subgroup analyses for dispensing outcome

Variable*	No (I:C)	Rate (95% CI)		Subgroup specific adjusted rate ratio (95% CI)‡	P value§
		Intervention (I)†	Control (C)†		
S5. Proportion of locums:					
<25%	54:41	0.147 (0.129 to 0.168)	0.157 (0.137 to 0.179)	0.937 (0.904 to 0.971)	0.005
≥25%	48:58	0.156 (0.135 to 0.180)	0.174 (0.151 to 0.200)	0.958 (0.925 to 0.993)	
S6. Proportion of nurses:					
<17%	81:72	0.154 (0.137 to 0.173)	0.165 (0.147 to 0.186)	0.921 (0.896 to 0.947)	<0.001
≥17%	62:74	0.149 (0.128 to 0.172)	0.149 (0.129 to 0.173)	1.051 (1.021 to 1.081)	
S7. Past dispensing rate:					
<18%	68:80	0.112 (0.098 to 0.129)	0.110 (0.096 to 0.126)	1.031 (1.001 to 1.063)	0.56
≥18%	76:69	0.182 (0.165 to 0.199)	0.194 (0.177 to 0.213)	0.992 (0.967 to 1.018)	
S8. Practice site¶:					
1 site	95:90	0.149 (0.129 to 0.172)	0.160 (0.139 to 0.185)	0.928 (0.903 to 0.954)	<0.001
≥2 sites	34:37	0.163 (0.141 to 0.188)	0.158 (0.137 to 0.182)	1.028 (0.990 to 1.067)	
S9. Follow-up completed before covid-19 pandemic:					
Yes	31:26	0.171 (0.138 to 0.212)	0.163 (0.131 to 0.202)	0.994 (0.955 to 1.036)	0.39
No	113:123	0.148 (0.130 to 0.167)	0.152 (0.134 to 0.172)	1.012 (0.991 to 1.035)	
S10. Level of deprivation:					
Low (rank** ≥14 387)	72:74	0.150 (0.134 to 0.167)	0.155 (0.139 to 0.173)	0.955 (0.929 to 0.981)	0.004
High (rank** <14 387)	72:75	0.154 (0.132 to 0.178)	0.150 (0.129 to 0.174)	1.061 (1.032 to 1.090)	

CCG=clinical commissioning group; CI=confidence interval.

All subgroup analyses were pre-specified in statistical analysis plan,^20^ although outcomes S8-S10 were introduced after trial started.

* Dichotomised on basis of median for continuous subgroups.

† Rates taken from random effects Poisson regression, incorporating CCG as random effect.

‡ Random effects Poisson regression, adjusting for baseline dispensing rate and incorporating CCG as random effect.

§ Taken from likelihood ratio test comparing random effects Poisson models with/without interaction term included, treating subgroup of interest as continuous variable where possible.

¶ Taken from practice follow-up questionnaire.

** Index of deprivation ranks every neighbourhood in England from 1 (most deprived area) to 32 844 (least deprived area).

### Comparison of hospital admission rates (co-primary outcome)

We found no difference in the rate of hospital admissions at 0.013 (0.010 to 0.018) and 0.015 (0.012 to 0.020) for the intervention and control arms, respectively. This translates into 13 or 15 admissions a year per 1000 children, and the rate ratio was 0.952 (0.905 to 1.003). As 1.003 lies below the 1.01 non-inferiority margin we set, the intervention was considered non-inferior. Pre-specified sensitivity analyses that incorporated hospital admissions with “missing diagnosis” did not change these results (supplementary table A). The seasonal winter peak of hospital admissions was absent during the pandemic (fig 2). The secondary outcome of emergency department attendance rates were 0.045 (0.038 to 0.054) and 0.044 (0.037 to 0.052) for the intervention and control arms, respectively. This translates into approximately 49 and 45 attendances a year per 1000 children; the rate ratio was 1.013 (0.980 to 1.047; P=0.44). Pre-specified sensitivity analyses that incorporated “missing diagnosis” admissions and emergency department attendances are shown in the supplementary material.

### Economic evaluation

We found no evidence of a difference in mean NHS costs (-£1999, –£6627 to £2630) in practices randomised to use the intervention compared with those that did not. This overall conclusion held under various sensitivity analyses, including a per protocol analysis.

## Discussion

This complex intervention, designed for use in pre-pandemic conditions, did not reduce overall dispensing of amoxicillin and macrolide antibiotics for children with respiratory tract infections presenting before and during the covid-19 pandemic. Neither did it increase the rate of hospital admissions for respiratory tract infection. Sensitivity and subgroup analyses showed decreased dispensing rates in the intervention arm for older children, practices restricted to one site, and practices with proportionally fewer nurse practitioners and in less deprived areas, although, given the number of sensitivity analyses carried out, these may have been chance findings. After the introduction of covid-19 restrictions in March 2020, average antibiotic dispensing halved in both study arms until the end of the trial. A post hoc sensitivity analysis suggested possible effectiveness of the intervention before the pandemic, although the magnitude of any effect was difficult to measure.

### Strengths and limitations of study

Strengths of the study include widespread geographical recruitment of general practices, rigorous study design and conduct, and near 100% completeness for the primary outcome. Using different recruitment strategies,^22^ and existing networks such as the clinical research networks, helped us to recruit some research naive practices and gave us practices located in more socioeconomically deprived areas, which mirrors the distribution of practices at the national level. Conducting the trial at the practice level removed the need for recruitment of patients and potential for differential recruitment between arms while focusing the clinician’s time on using the intervention, reflecting real life practice. Dispensed antibiotics is a good measure of antibiotic use in that it reflects parental as well as clinicians’ perception of the need for an antibiotic: “delayed” and “immediate” prescriptions will not be collected by unconvinced parents.

Limitations of the study include that the March 2020 and subsequent covid-19 lockdowns meant a wholesale change from face-to-face to remote consultations in primary care, as well as greatly reduced capacity to support non-covid research. The CHICO intervention was designed for use in face-to-face consultations, and clinicians may have been reluctant to use it remotely. Secondly, recorded intervention use was low in relation to the likely number of 0-9 year old children in whom it could have been used; interviews with clinicians suggested that they often used the tool for “borderline” cases, and usage also dramatically fell after March 2020. We estimated use assuming that the dispensed items were all for respiratory tract infection consultations and that 50% of the children consulting were given these. The dispensing rate in the intervention arm was 15.5 items per 100 children; given that the median number of 0-9 year olds was 975 children in 144 intervention practices, this yields 21 762 dispensed items and 43 524 consultations for respiratory tract infection. This is speculative but would suggest that the 11 944 uses of the intervention was around one in every four consultations. This could have been higher if clinicians were able to determine prognostic risk group by memory. Thirdly, less than half of the eligible CCGs approached took part in the study; the difficulty in contacting some CCGs or lack of response makes assessing what bias this may have introduced difficult, although we did have at least one CCG in each of the 15 clinical research network areas in England. Fourthly, just over a quarter of the practices that expressed an interest and met the eligibility criteria subsequently declined, citing lack of capacity or failing to return forms. The impact of the pandemic may have played a part, but this questions the generalisability of the findings. Fifthly, around 2.5% of practices close or merge each year in England, and the number of multiple site practices was larger than anticipated (28%), substantially increasing the number of registered patients. which has implications for cluster trials and sample size calculations. The contrast in antibiotic dispensing rates between arms in multiple site practices compared with single site practices was notable and questions whether a single practice champion for multiple sites was able to promote intervention use effectively. Sixthly, a further limitation was use of the number of children aged 0-9 registered at the practice as the denominator rather than the number of children consulting for respiratory tract infection. A detailed record of how many children consulted for respiratory tract infection and whether the intervention was used would have been difficult to obtain, but its absence limits our ability to comment on whether the intervention was used as planned or whether a lack of use affected our findings. Finally, general advice is that algorithms should be externally validated before an evaluation of clinical effectiveness. However, this was not possible given the absence of a suitable dataset.

### Comparison with other studies

A similar trial randomising general practices to receive a decision support tool embedded in electronic medical records with the aim of reducing antibiotic prescribing for respiratory infections in a wider age range found evidence that the intervention was effective in adults (15-84 years).^23^ However, as with our study, it did not find evidence of reduced prescribing in children (0-14 years), nor in adults aged >84 years. We speculate that this could be because clinicians and/or parents are more risk averse and unwilling to withhold antibiotic treatment when managing children rather than adults. This study also monitored safety by reporting hospital admissions for serious bacterial infections, finding no evidence that these were increased in the intervention group. The reduction in rates of consultation and antibiotic dispensing that we observed between pre-pandemic and covid-19 pandemic periods is consistent with that reported in several other studies, both in the UK and globally.^24^ ^25^ ^26^

### Implications for clinical practice and future research

We did not find evidence to support the widespread use of the intervention, but our subgroup and sensitivity analyses suggest that the intervention may hold promise in some clinical groups. Our post hoc analyses were underpowered but provided a signal that the intervention held some promise under non-pandemic conditions. Future research should seek to confirm these signals and develop interventions tailored to the clinical needs of nurses, larger sites, younger children, and socioeconomically deprived communities, in post-pandemic conditions.

### Conclusions

Embedding a multifaceted intervention into general practice for children presenting with acute cough and respiratory tract infection did not reduce antibiotic dispensing or affect hospital attendance for respiratory tract infections. Remote consulting during the pandemic may have affected the effectiveness of the intervention. More research is needed to confirm potential effects seen in practices with fewer prescribing nurses, restricted to a single site, or located in areas of lower deprivation and in younger children and post-pandemic conditions.

## What is already known on this topic

Inappropriate use of antibiotics needlessly contributes to antimicrobial resistance
Children with acute cough and respiratory tract infection, usually with viral aetiology, are the most frequent patient group seen in primary care, with up to half treated with antibiotics
Uncertainty about poor prognosis is an important driver of indiscriminate antimicrobial use in children

## What this study adds

The multifaceted intervention investigated included eliciting parental concerns, an algorithm to identify children at very low risk of hospital admission, and safety netting advice for carers
The intervention, designed for pre-pandemic conditions, did not reduce overall antibiotic dispensing or increase respiratory tract infection related hospital admissions
Subgroup and sensitivity analyses suggested that the intervention may be effective for older children and practices with fewer prescribing nurses, on a single site, or located in less deprived areas

## Data Availability

Data may be obtained from a third party (clinical commissioning groups and Public Health England) and are not publicly available.

## Web extra

Extra material supplied by authors

Web appendix: Supplementary materials

## Notes

Contributors: AH, PSB, PJL, NAF, and JI were responsible for developing the research questions. PSB, AH, JI, PJL, CCabral, CClement, EB, MG, JH, SC, JAL, and NAF were responsible for the study design and collection of data. PS, SB, and JT were responsible for study management and coordination. GY, PD, and CClement were responsible for the analysis of the data. PB drafted the paper and is the guarantor. All authors read, commented on, and approved the final manuscript. The corresponding author attests that all listed authors meet authorship criteria and that no others meeting the criteria have been omitted.

The lead author affirms that the manuscript is an honest, accurate, and transparent account of the study being reported; that no important aspects of the study have been omitted; and that any discrepancies from the study as originally planned (and, if relevant, registered) have been explained.

Dissemination to participants and related patient and public communities: Patients and clinicians were involved in the design, conduct, reporting, and dissemination plans of this research. Refer to the Methods section for further details. Participants will be contacted with a summary of the findings when the embargo for the published main findings is lifted.

Provenance and peer review: Not commissioned; externally peer reviewed.
