# Supplementary material for: Multi-faceted intervention to improve management of antibiotics for children presenting to primary care with acute cough and respiratory tract infection (CHICO): efficient cluster randomised controlled trial
Source: BMJ. 2023 Apr 26;381:e072488. doi: 10.1136/bmj-2022-072488 (PMC10131137; doi:10.1136/bmj-2022-072488)

Supplementary material

Methods

Sensitivity analyses for the dispensing outcome

To test the robustness of the dispensing primary outcome several sensitivity analyses were carried out:

(1) The first of these was a per protocol analysis, which aimed to exclude non-compliers from the intervention arm. Compliance was pre-specified, in the statistical analysis plan, as the number of uses divided by the list size. A practice was considered 'compliant' if this value was ≥0.05. If compliance couldn't be calculated, the practice was excluded. Practices that merged were also excluded, if they merged with a practice in the control arm.
(2) The source routine data on dispensing were separated into two figures, one for children aged 0-4 and one for children aged 5-9. The figures were combined in the primary analyses, to look at the dispensing rate for all 0-9 year olds. In sensitivity analyses, the figures were kept separate, to assess the treatment effect in each age epoch group.
(3) Data was captured on dispensed items in children aged 0-9 and those with an 'unknown age'. A worst case scenario approach included all 'unknown age' dispensed items as age 0-9.
(4) Another sensitivity analysis excluded practices that had taken part in the pilot phase of the study, as some of the documents used during this phase (e.g. FAQ document) were altered for the main trial.
(5) Given the impact of Covid-19 on dispensing rates an additional sensitivity analysis added a continuous variable for the number of months of follow up that were on, or after, March 2020 (both intervention and control arms).
(6) Post-hoc: After the conducting the per protocol analysis, it became clear that the method was biased and that a complier-average causal effect (CACE) model may be more appropriate. This involved an instrumental variable analysis method (using random allocation as the instrumental variable) of the effect of the trial intervention in those complying, or who would have complied, with the intervention; using a generalized method of moments estimator.
(7) Post hoc: Another included all practices with at least one follow up month before March 2020 and excluded any months on, or after, March 2020. Utilising the number of months included as the denominator (e.g. 0.8 if 10 months included) and the number of items dispensed in the months prior to March 2020 as the numerator.
(8) Post hoc: In the primary analysis both amoxicillin and macrolide items were included as dispensed antibiotics items. In a sensitivity analysis macrolides were excluded.
(9) Post hoc: The CCG level was incorporated as a random effect to allow for the 'shared' working practices within practices in a CCG. In a post-hoc sensitivity analysis used the Primary Care Network (PCN) as the random effect instead.
(10) Post hoc: There were also some intervention practices that had unexpected delays in importing the intervention (mostly due to Covid-19). This was accounted for in a sensitivity analysis, by adding a continuous variable for the number of months the practice delayed their start date, after randomisation.

Sensitivity analyses for the hospitalisation and A&E attendance outcomes

As the hospitalisation and A&E data were collected from CCGs, there were various assumptions that were made. To test the robustness of the hospitalisation primary outcome and A&E secondary outcome, several sensitivity analyses were carried out. For hospitalisation and A&E routine data, diagnosis codes are sometimes missing.

(1) Using the proportion of LRTI attendances out of those with a diagnosis we could then deduced the proportion of "diagnosis missing" attendances that were likely to be attributable to RTI. These were added to the LRTI diagnosis figures in a sensitivity analysis.
(2) As a 'worst case scenario' approach, all 'missing diagnosis' hospitalisations/attendances were included in separate sensitivity analyses.
(3) As with the dispensing outcome, the team added a sensitivity analysis that would add a COVID-19 time variable, ranging from 0-12, to account for the months of follow up affected by COVID-19 (on or after March 2020). This variable was added as a covariate in a sensitivity analysis.
(4) Complete data were provided for 46 out of 47 CCGs, while 1 CCG only provided suppressed monthly data. Therefore, their annual data (summations of n<5) may be a misrepresentation of the true figure and their data were removed in a sensitivity analysis.

Results

Sensitivity analyses for the hospitalisation and A&E attendance outcomes

Including a proportion of the 'missing diagnosis' hospitalisations/A&E attendances, as RTI related, gave very similar results to the primary and secondary outcomes (Table A and B). However including all of 'missing diagnoses' as LRTI related, led to different results. For hospitalisations, it just tipped the balance of non-inferiority with a confidence interval of 0.920, 1.106. For A&E attendances, including missing diagnoses led to higher rates in the intervention arm. Including a variable from 1-12, to account for the number of months affected by COVID-19, provided results that agreed with the primary and secondary outcome results. As did the removal of the single CCG without annual data.

Table A. Sensitivity analyses for the hospitalisation outcome

	Intervention Rate[a] (95% CI)	Control Rate[a] (95% CI)	Adjusted RR (95% CI)[b]
Primary analysis	0.013 (0.010, 0.018)	0.015 (0.012, 0.020)	0.952 (0.905, 1.003)
Including % missing diagnoses[c]	*0.013 (0.010, 0.018)*	*0.015 (0.012, 0.020)*	*0.950 (0.903, 1.000)*
Including all missing diagnoses[d]	*0.014 (0.011, 0.018)*	*0.016 (0.012, 0.021)*	*0.967 (0.920, 1.016)*
Covid-19 month indicator[e]	*0.013 (0.010, 0.018)*	*0.015 (0.012, 0.020)*	*0.936 (0.889, 0.986)*
Excluding single CCG[f]	*0.013 (0.010, 0.173)*	*0.014 (0.011, 0.019)*	*0.951 (0.900, 1.004)*

Table B. Sensitivity analyses for the A&E rate outcome

	Intervention Rate[a] (95% CI)	Control Rate[a] (95% CI)	Adjusted RR (95% CI)[b]	P value
Secondary analysis	0.045 (0.038, 0.054)	0.044 (0.037, 0.052)	1.013 (0.980, 1.047)	0.437
Including % missing diagnoses[c]	*0.053 (0.045, 0.064)*	*0.052 (0.043, 0.062)*	*1.006 (0.977, 1.037)*	*0.680*
Including all missing diagnoses[d]	*0.104 (0.086, 0.126)*	*0.104 (0.086, 0.126)*	*0.976 (0.956, 0.996)*	*0.017*
Covid-19 month indicator[e]	*0.045 (0.038, 0.054)*	*0.044 (0.037, 0.052)*	*1.013 (0.980, 1.047)*	*0.445*

Excluding single CCG[f]	*0.046 (0.038, 0.054)*	*0.043 (0.036, 0.052)*	*1.021 (0.987, 1.056)*	*0.226*

Footnote: P values were not presented in Table S1a as this was a non-inferiority outcome, [a]Rates taken from a random effects Poisson regression, incorporating CCG as a random effect, [b]Random effects Poisson regression, *adjusting for baseline hospitalisation/A&E attendance rate* and incorporating the CCG as a random effect, [c]A proportion of hospitalisations/A&E attendances with a missing diagnosis were included as RTI diagnoses: (RTI events/total events)*missing diagnosis events, [d]All hospitalisations/A&E attendances with a missing diagnosis were included a RTI diagnoses, [e]Including a numerical variable (0-12) to indicate how many months were affected by COVID-19, [f]Excluding one CCG (I=4, C=5), who did not provide annual data

Figure A. Sub group analyses for the dispensing primary outcome, for practices in the intervention arm (orange diamonds) and control arm (blue circles)

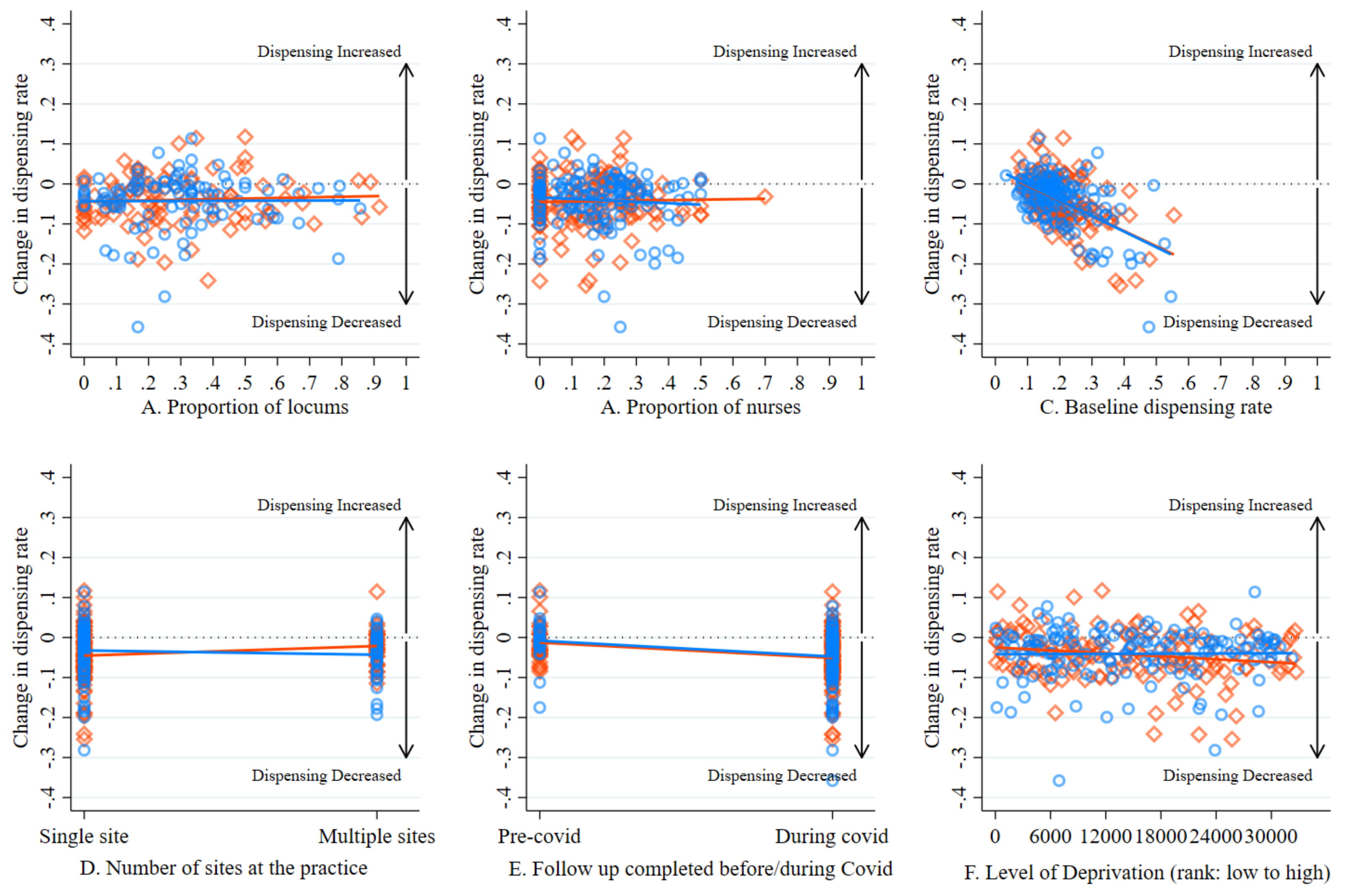

Supplement: Supplementary file 1 — Web appendix: Supplementary materials [file blap072488.ww1.pdf]
